# Systematically profiling and annotating long intergenic non-coding RNAs in human embryonic stem cell

**DOI:** 10.1186/1471-2164-14-S5-S3

**Published:** 2013-10-16

**Authors:** Xing Tang, Mei Hou, Yang Ding, Zhaohui Li, Lichen Ren, Ge Gao

**Affiliations:** 1 School of Life Sciences, State Key Laboratory of Protein and Plant Gene Research, Center for Bioinformatics, Peking University, Beijing, 100871, P.R. China; 2 School of Life Sciences, Tsinghua University, Beijing, 100084, P.R. China

## Abstract

**Background:**

While more and more long intergenic non-coding RNAs (lincRNAs) were identified to take important roles in both maintaining pluripotency and regulating differentiation, how these lincRNAs may define and drive cell fate decisions on a global scale are still mostly elusive. Systematical profiling and comprehensive annotation of embryonic stem cells lincRNAs may not only bring a clearer big picture of these novel regulators but also shed light on their functionalities.

**Results:**

Based on multiple RNA-Seq datasets, we systematically identified 300 human embryonic stem cell lincRNAs (hES lincRNAs). Of which, one forth (78 out of 300) hES lincRNAs were further identified to be biasedly expressed in human ES cells. Functional analysis showed that they were preferentially involved in several early-development related biological processes. Comparative genomics analysis further suggested that around half of the identified hES lincRNAs were conserved in mouse. To facilitate further investigation of these hES lincRNAs, we constructed an online portal for biologists to access all their sequences and annotations interactively. In addition to navigation through a genome browse interface, users can also locate lincRNAs through an advanced query interface based on both keywords and expression profiles, and analyze results through multiple tools.

**Conclusions:**

By integrating multiple RNA-Seq datasets, we systematically characterized and annotated 300 hES lincRNAs. A full functional web portal is available freely at http://scbrowse.cbi.pku.edu.cn. As the first global profiling and annotating of human embryonic stem cell lincRNAs, this work aims to provide a valuable resource for both experimental biologists and bioinformaticians.

## Background

The great potential of human embryonic stem cell (hES) in clinical usage inspired scientists to investigate underlying mechanisms for their unique pluripotency and self-renew characteristics [1-9]. Recently, several studies demonstrate that long intergenic non-coding RNAs (lincRNAs) play key roles in maintaining pluripotency [10,11], modulating reprogramming [12] and differentiation [13]. Knockdown of multiple lincRNAs has great effect on global gene expression pattern and could cause exit from the pluripotent state [10]. Several human lincRNAs are further showed to be involved in core regulatory feedback circuits of hES cells and directly regulated by well-known key pluripotency transcription factors such as Oct4 and Nanog [10,14,15]. As more and more human lincRNAs were identified [9,10,13-16], systematically characterizing human embryonic stem cell lincRNAs will not only shed lights on the hES transcriptome dynamics but also help revealing biological functions of these novel regulators.

Combining a comprehensive collection of human embryonic stem cell RNA-Seq datasets with Human BodyMap 2, we validated that 295 previously annotated lincRNAs were expressed in multiple human embryonic stem cell samples and further identified five novel hES lincRNAs through de novo assembling. Global statistical analysis revealed that these lincRNAs' expression levels are lower than that of their protein-coding counterparts. Functional analysis further demonstrated that hES lincRNAs were preferentially involved in multiple development processes including embryo development, ribosome biogenesis, and aging. To help explore the abundant information effectively, we built an integrative web portal for scientists to browse, search and perform analysis of all lincRNAs through an intuitive Web interface. It could be accessed freely at http://scbrowse.cbi.pku.edu.cn.

## Results

### 300 lincRNAs are transcribed in human embryonic stem cells

In order to systematically profile hES lincRNAs, we firstly compiled a known human lincRNA catalog by integrating multiple public sources. Annotated lincRNA gene models were extracted from Ensembl, UCSC and RefSeq. Redundant gene models were identified and merged based on the genomic coordinates, resulting in 5,571 standalone annotated lincRNA genes (See Methods and Materials, as well as the Additional File 1 for more details). Moreover, we surveyed and manually screened published hES RNA-Seq datasets in several public repositories, resulting in a list of 31 wild-type human embryonic stem cell samples. Out of which, 19 high-quality datasets with at least 50nt read length were further selected for follow-up analysis to minimize technological biases caused by early Solexa platforms (see Additional File 2 for more details). In addition, transcriptome profiling for 16 adult normal tissues derived from Illumina Human BodyMap 2 Project were also incorporated as control.

To find novel hES lincRNAs, we performed *de novo *assembling against all wild-type hES samples. After excluding annotated lincRNAs and non-lincRNA transcripts (e.g. known protein-coding genes, miRNAs and tRNAs), five novel lincRNAs were eventually identified. Combining with previous annotated catalog, we got a full list with 5,576 human lincRNA genes (5,571 known lincRNAs and 5 novel ones, see Methods and Materials for more details)

**Figure 1:**
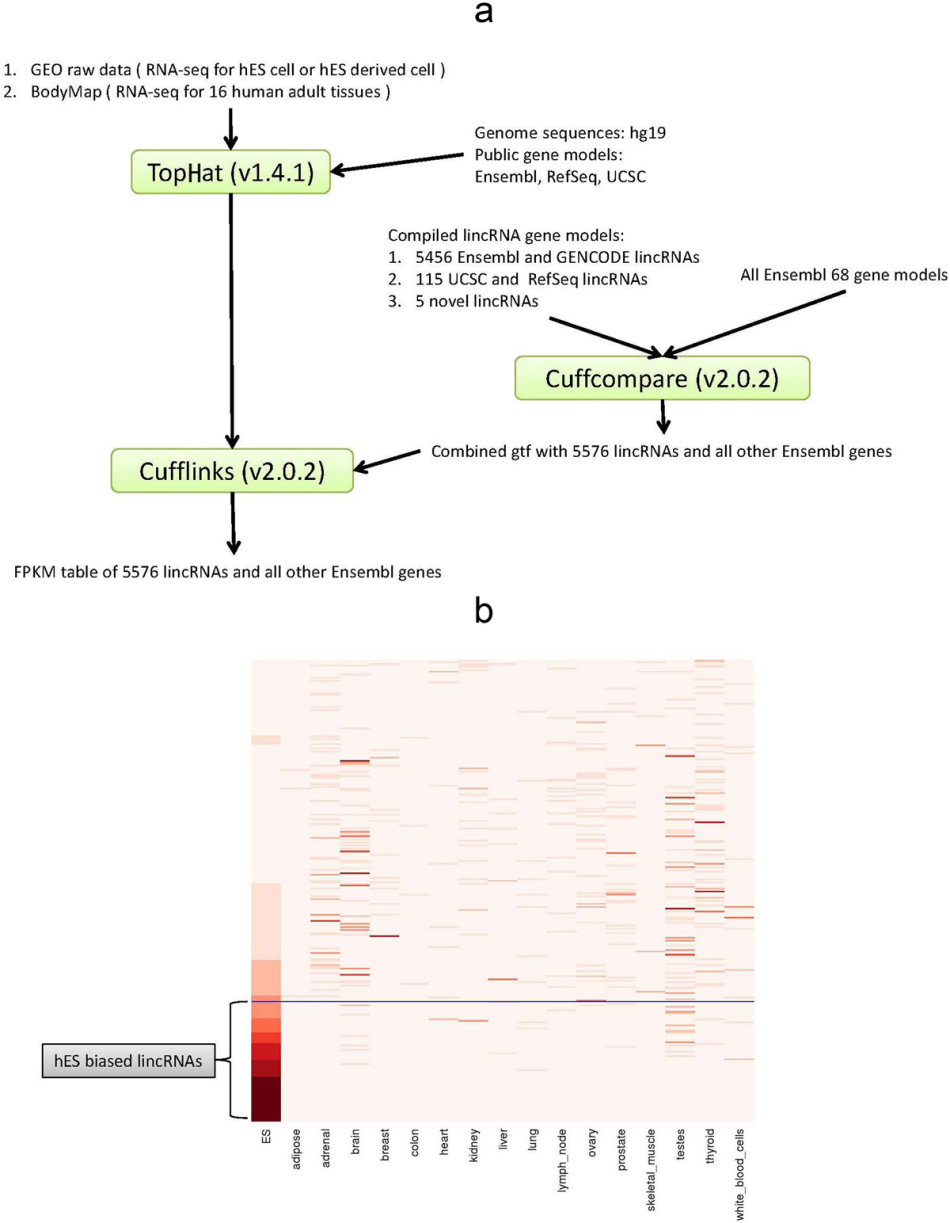
**300 hES lincRNAs**. (a) The analysis pipeline. We mapped reads onto hg19 human genome using TopHat (v1.4.1) [39] with a reference helped strategy in case of failure to map some junction reads. We merged gene models from different resources using cuffcompare, and calculated gene expression level using Cufflinks (v2.0.2) [17] based on gene models we compiled. (b) Abundance of 300 hES lincRNAs across hES and human adult tissues. Color intensity represents the fractional density across the row of FPKM as estimated by Cufflinks [17]. Classifying by tissue specificity index [46], one fourth (78 out of 300) hES lincRNAs were biasedly expressed in hES (tau > 0.9).

We then estimated their expression levels across 19 wild-type hES samples and 16 normal adult tissue samples with the standard FPKM (Fragments Per Kilobase of transcript per Million mapped reads) index [17] (Figure 1a). In case of over-representation of hES samples, we took the median values as a representative expression index. Noticeably, only one third (1,826 out of 5,576) lincRNAs were found to be expressed in at least one tissue (i.e. FPKM >= 1), much lower than protein-coding genes (Single-tailed Fisher's exact test, odds ratio = 0.058, p-value < 2.2e-16), suggesting a higher temporal-space expression specificity of lincRNAs than of protein-coding ones [18,19].

300 (~16.43%) of the expressed lincRNAs were detected as being expressed in hES (Figure 1b). Open chromatin marks were detected to be significantly enriched at their promoters (Fisher's exact test, H3K4me3, p-value < 2.2e-16; H3K4me2, p-value < 2.2e-16) and gene bodies (Wilcoxon test, H3K36me3, p-value<2.2e-16; H3K79me2, p-value< 2.2e-16), confirming active transcription of these genomic regions in human ES cells [20,21] (Additional File 3).

*Nanog *and *Oct4 *are both well-known essential transcription factors in hES [6,22,23]. The promoters of hES lincRNAs were found to be enriched with binding sites of *Nanog *(Fisher's exact test, adjusted p-value < 2.2e-16, odds ratio = 5.0) but not *Oct4 *(adjusted p-value = 1, odds ratio = 1.7), suggesting different regulation of lincRNAs between these two pluripotency factors in hES.

Comparing with other lincRNAs, hES lincRNAs generally had more complex transcript structure with longer transcript length (1,215 nt versus 906 nt, p-value < 2.2e-16), more exons per transcript (3.44 versus 3.00, p-value = 2.2e-06) and more alternative isoforms per gene (2.76 versus 1.42, p-value < 2.2e-16). On the other hand, being consistent with previous reports [18,19], both their expression level and breath were overall lower than of their protein-coding counterparts.

### Systematic annotation of hES lincRNAs

**Figure 2:**
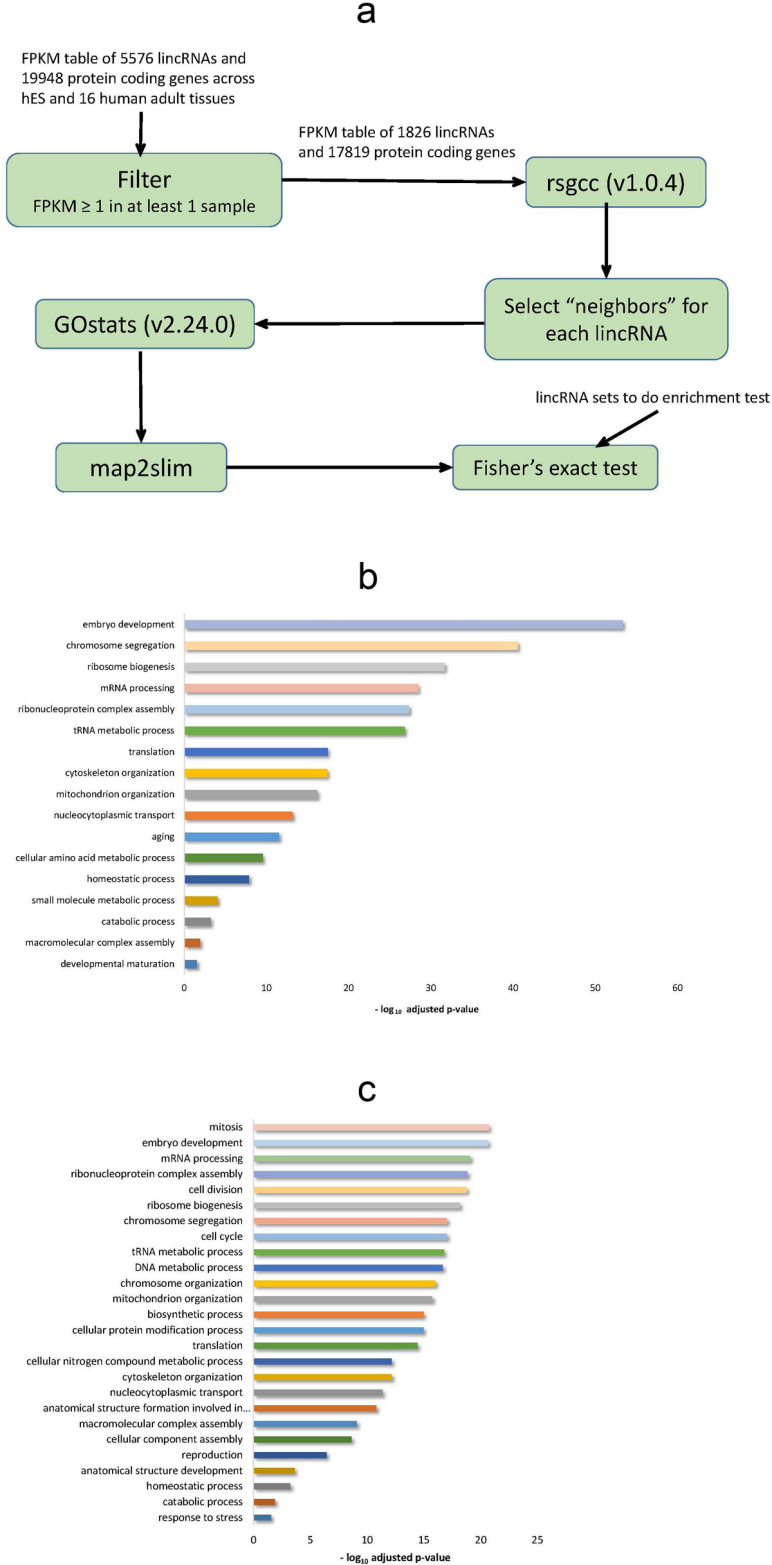
**Function annotation of human lincRNAs**. (a) flowchart of co-expression association annotation. Expression profile across hES and 16 human adult tissues was filtered for low expressed genes. Then, Gini correlations were calculated and neighbors of each lincRNA were selected to do GO enrichment analysis. We got a list of enriched GO slim terms and their corresponding lincRNAs by mapping GO terms to GO slim terms. Finally, we did Fisher's exact test to test whether interested lincRNA sets were enriched in each GO slim terms. Please see details in GO enrichment analysis section in Data Preparation of Methods and Materials. (b) Significantly enriched biological processes of hES lincRNAs, (c) hES biased lincRNAs.

Largely due to the elusive nature of lincRNA functional mechanism, it's still hardly practical to infer functions of lincRNAs from their nucleotide sequences solely [24]. Thus, we tried to annotate lincRNAs based on co-expression association strategy [15,25]. In brief, for each lincRNA, we firstly identified protein coding genes with strong expression correlation ("neighbors"), and assigned the corresponding Gene Ontology (GO) terms of these neighbors as the annotations of this lincRNA. To get an overview, fine-grained terms were further projected onto generic Gene Ontology slim (GO slim) terms (see Methods and Materials for more details). Finally, we successfully annotated more than 96% (1,765 out of 1,826) expressed lincRNAs (Figure 2a).

Partly due to their expression specificity, a bit lower proportion (93.3%, 280 out of 300) of hES lincRNAs were annotated. Global statistical analysis suggested that these hES lincRNAs were preferentially involved in embryo development (108 lincRNAs, p-value = 4.56e-54) and ribosome biogenesis (41 lincRNAs, p-value = 2.10e-32). On the other hand, hES biased lincRNAs were more likely to be involved in mitosis (67 lincRNAs, p-value = 1.90e-21), cell cycle (72 lincRNAs, p-value = 8.96e-18), reproduction (52 lincRNAs, p-value = 3.53e-7), as well as embryo development (76 lincRNAs, p-value = 2.13e-21) and ribosome biogenesis (40 lincRNAs, p-value = 6.86e-19) (Figure 2b and Figure 2c). Interestingly, the five *de novo *assembled hES lincRNAs were involved in embryonic epithelial tube formation and regulation of cell cycle, suggesting their putative important roles.

In addition to functional annotation, we further investigated the evolutionary pattern of hES lincRNAs. Previous studies have shown that the exon sequences of lincRNA are less conserved than that of protein coding genes, while more conserved than neutrally evolving ancestral repeat sequences [18,19]. On the other hand, despite of their relatively rapid sequence turn-over rate, several lincRNAs have been reported to have homologs in remote species, suggesting distinct evolutionary patterns among different classes of lincRNAs [18,19,26,27].

**Figure 3:**
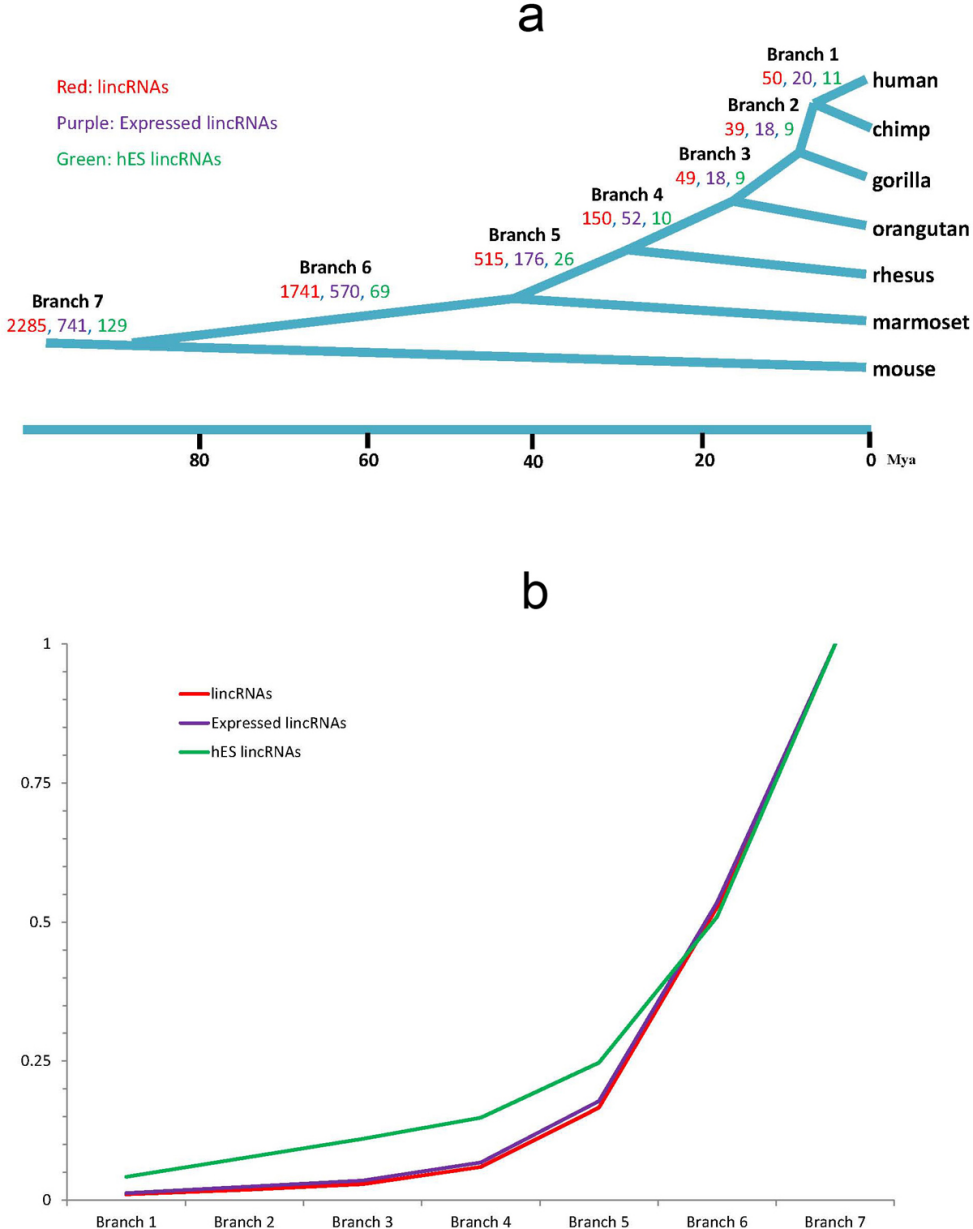
**Evolutionary dating of lincRNAs**. (a) The counts of lincRNAs originated from each branch (Red: all lincRNAs; Purple: expressed lincRNAs; Green: hES lincRNAs). (b) Cumulative distribution of age distributions of the three lincRNA gene sets.

Based on the genome alignment from UCSC, we searched homologs across multiple mammalian genomes including five representative primates (chimp, gorilla, orangutan, rhesus and marmoset) and one rodent (mouse). The origination branch of lincRNAs was further inferred (i.e. "dated") in the mammalian tree following parsimony principle (see Methods and Materials for details). Finally, we dated 86.60% (4,829 out of 5,576) human lincRNAs onto different evolutionary branches (Figure 3a).

For hES lincRNAs, 263 out of 300 were successfully dated. Around half (129 out of 300) were dated at the root of the mammalian tree, i.e. being conserved across mouse and human lineage. A close look of the respective mouse homologous regions found that more than 80% (109 out of 129) of them had at least one active histone markers (H3K9ac, H3K27ac, H3K36me3, and H3K4me3) and only two of them had repressive histone modifications (H3K9me3, H3K27me3) in mouse ES cells, with one third (33) also posed directly upstream PolII binding measured by ChIP-Seq assay, suggesting their *bona fide *mouse ES expression.

To our surprise, hES lincRNAs were found to be generally younger than other lincRNAs (Wilcoxon test, p-value = 4.46e-07) (Figure 3b). Consistently, we also found that human specific lincRNAs were more likely to be hES lincRNAs (Single-tailed Fisher's exact test, odds ratio =6.3, p-value = 0.0054). Eleven hES lincRNAs were identified as human-specific. Similar to previous reports [28], we also found these human-specific hES lincRNAs more likely to hold HERVH transposable elements (TEs) than mouse-conserved ones (Single-tailed Fisher's exact test, odds ratio= 66.3, p-value = 0.0001). Of interest, two of them (ENSG00000228437, ENSG00000254339) were found to be under strong intra-population purifying selection indicated by low derived allele frequency (< 0.1) [29], suggesting their potential human-specific functions.

### Integrative web portal for visualizing and analyzing data

To facilitate further investigation of these hES lincRNAs, we integrated gene models and annotations with multiple related biological data into an integrative web portal. Powered by ABrowse [30], the portal aims to providing users a fully interactive environment for browsing, searching and analyzing these lincRNAs as well as annotations through an intuitive interface.

**Figure 4:**
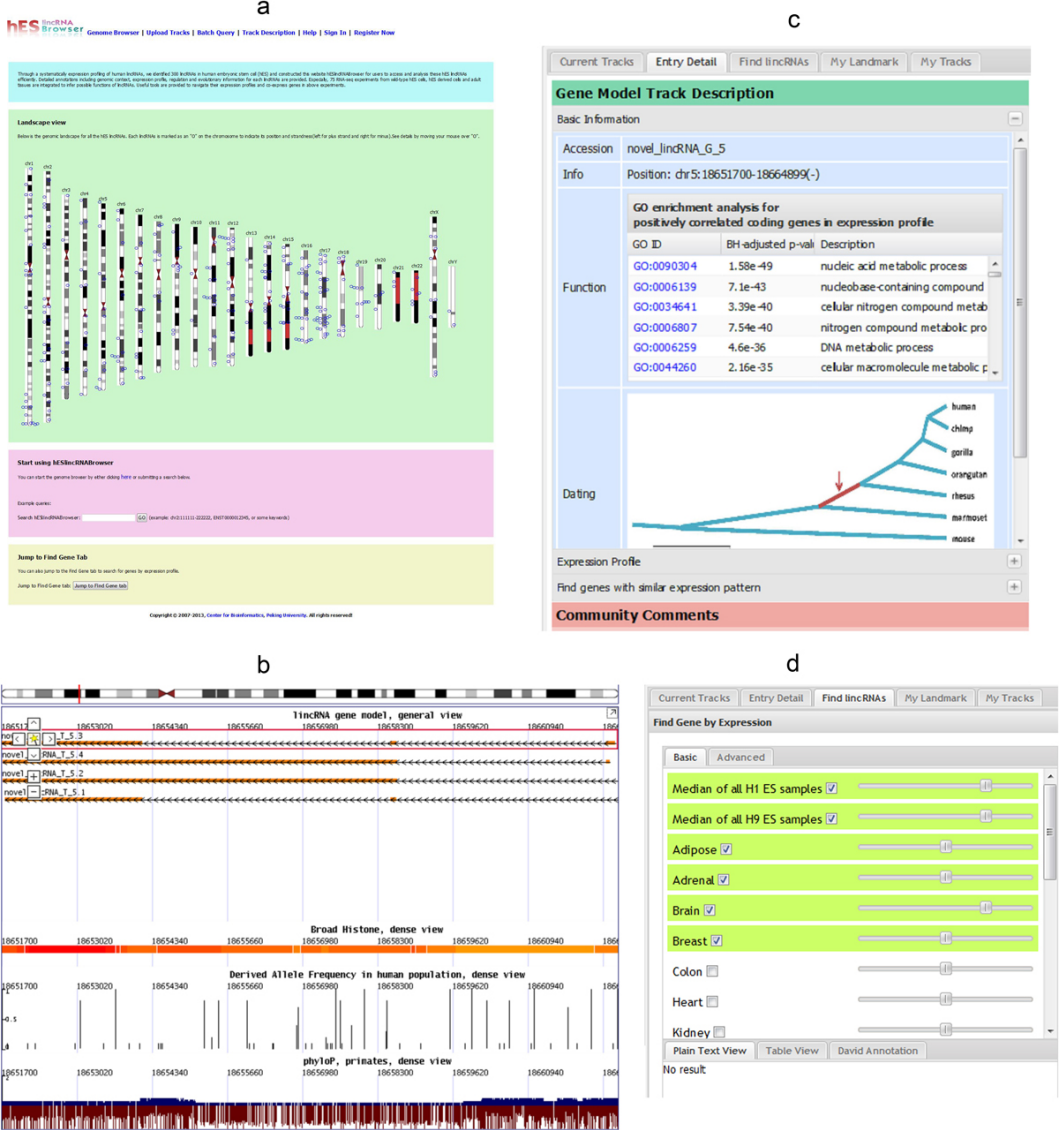
**Web portal interface**. (a) Welcome page; (b) main canvas for genome browse; (c) control panel to see detailed annotations; (d) find lincRNAs through user-specified expression pattern.

User can start his/her navigation by either choosing a hES lincRNA on the chromosome map, or jumping to interested genes directly (Figure 4a). The main interface is presented as a typical genome browse, with heading navigation bar, control panel (at the left) (Figure 4c) and the main browsing canvas (Figure 4b). Multiple tracks could be displayed simultaneously after turning them on in the "Tracks" box. Currently, twelve tracks covering gene model, transcription regulation and comparative genomics are available (see http://scbrowse.cbi.pku.edu.cn/tutorial/index.jsp for detailed descriptions for all tracks).

The gene model tracks are designed to present both gene structures and annotations. In addition to the complete list of 5,576 human lincRNAs (track "lincRNA gene model"), protein-coding genes from Ensembl 68 (track "protein coding gene model"), two human lincRNA sets from LNCipedia [31], Cabili *et al. *[18] and one hES long non-coding RNA (lncRNA) catalog from Sigova *et al. *[32] are also available as references. Detailed annotations for each record will be shown in the "Entry detail" tab (of left-side Control panel, Figure 4c) after a click.

For each identified human lincRNA, multiple annotations are grouped as three sheets in the "Entry detail" tab:

1) "Basic Information" sheet contains its genomic location, original accession number and the source link, as well as the functional and evolutionary annotations.

2) "Expression Profile" sheet shows global expression profile of the given lincRNA across multiple samples. To get a comprehensive view, we also incorporated 24 differentiated embryonic stem cell samples in addition to the 31 wild-type embryonic stem cell samples and 16 normal adult human tissues. After choosing samples in the "Sample Tree", the user could visually inspect the expression profile through a bar chart, and add mean expression for similar samples (i.e. samples in the same group) if needed.

3) The last sheet ("Find genes with similar expression pattern") allows user to identify co-expressed genes quickly. After specifying the correlation coefficient measurement (Pearson's r, Spearman's rho or Gini correlation coefficient), the cut-off and the samples, user can either view the matched genes interactively, or download in batch for further analysis.

To offer insights for the regulation and evolution of hES lincRNAs, we further integrated additional tracks derived from several public annotation sources. Multiple transcription factor binding and histone modification tracks, including more than 50 different transcription factors and 11 different types of histone modifications in H1 hES cell line [33] are grouped as "Transcriptional Regulation". Similarly, evolutionary conservation tracks covering both inter-species (measured by PhloyP score [34]) and intra-species (measured by derived allele frequency [29]) comparisons are grouped as "Evolution". All these tracks could be easily turned on or off through the "Tracks" box.

In addition to rich annotations, the portal also provides powerful searching tools for users to find lincRNAs quickly. In addition to common text-based search and sequence-based search (through the "Advanced Search" box), the portal allows users to search lincRNAs with specified expression pattern through the dedicated "Find lincRNAs" tab in the Control panel (Figure 4d). With an intuitive interface, the user can specify the expression pattern across multiple samples by either interactively "drawing" in the "Basic" sub-tab or inputting numbers precisely in the "Advance" sub-tab. Similar to the output of "Find genes with similar expression pattern" sheet, the result could also be exported as plain text for further processing (Figure 4d).

Furthermore, multiple utilities were implemented to make it easier for users to analyze data through the portal. After registration, the user can upload customized tracks and manage them through the "My Tracks" tab in the Control panel. Even more conventionally, the registered user can add Instant Note for any genomic region on-the-fly by clicking-and-dragging. All these user generated annotations could be seamlessly integrated with existing tracks, and freely set as "public" or "private" when necessary.

## Methods

### Compiling annotated human lincRNA catalog

To get a comprehensive annotated human lincRNA catalog, we firstly extracted all annotated lincRNA gene models from Ensembl (v68), then filtered 6,730 RefSeq NR_ records and 10,654 UCSC noncoding genes based on multiple criteria. Inspired by previous studies [18,19], we took a conservative strategy. Briefly, only multi-exon transcripts satisfying 1) length >200bp, 2) not overlapped with known genes, and 3) classified as "non-coding" by CPC [35] were kept for further analysis. Redundant gene models were identified and merged based on genomic coordinate, resulting in 5,571 standalone annotated lincRNA genes (see also Additional File 1 for details).

### RNA-Seq data collection

We manually screened all Illumine human RNA-Seq datasets in NCBI GEO [36,37]. In case of potential genomic contamination, only samples with polyA plus libraries were kept. Eventually, we got a list of 31 wild-type samples (19 H1, 4 H9 and 8 other hES cell lines) and 24 differentiated embryonic stem cell samples. To minimize technological biases caused by early Solexa platforms, we further chose 19 high-quality wild-type datasets with at least 50nt read length for follow-up analysis (see Additional File 2 for more details). In addition, transcriptome profiling for 16 adult normal tissues generated by Human BodyMap 2 Project were also incorporated.

### De novo assembling lincRNAs from RNA-Seq data

Following standard protocol [38], we mapped raw reads of the 19 high quality wild-type hES samples onto the human reference genome (hg19) by TopHat [39] and assembled mapped reads into transcripts by Cufflinks [17]. All assembled transcripts were firstly filtered using similar criteria being described above, and transcripts with low expression level were further removed to control false positives due to transcription noises (see Additional File 4 for more details).

Finally, after combining with previous annotated catalog, we got a full list with 5,576 human lincRNA genes (5,571 known lincRNAs and 5 novel ones).

### Identifying hES lincRNAs

Several efforts have been taken to ensure the quality of hES lincRNAs catalog. For effectively reducing false positives caused by random "bench effect", we systematically screened multiple heterogeneous datasets generated by different labs around the world (Figure 1).

To further improve the robustness, we applied a stringent criteria (median FPKM across multiple human ES samples > 1) when calling hES lincRNAs. On the other hand, a rather loose cut-off (0.07), was used by Sigova et al. [32], resulting in large numbers of marginally expressed candidates. In fact, more than 80% (2,910) of the Sigova et al. reported hES long noncoding RNAs (3,548) have low expression levels (< 1), and less than 18% (638 out of 3,548) Sigova's hES long noncoding RNAs could pass our filter, including only 37 (less than 10%) Sigova's distant hES long noncoding RNAs.

### ChIP-Seq analysis for transcription factors and histone modifications

Respective ENCODE ChIP-seq datasets for both human and mouse ES cell lines were downloaded (from http://hgdownload.cse.ucsc.edu/goldenPath/hg19/encodeDCC and http://hgdownload.cse.ucsc.edu/goldenPath/mm9/encodeDCC). Replicates were merged firstly and then feed into MACS [40] for peak calling with p-value cutoff = 1e-10.

The upstream 3kb and downstream 1kb of annotated transcript starting sites were defined as promoter regions [41]. TFBS or promoter biased histone modifications as H3K4me3, H3K4me2, H3K27me3 and H3K9me3 located within these regions are considered as effective regulatory sites.

### Gene ontology (GO) enrichment analysis

According to previous evaluation [42], we calculated the Gini correlation coefficient (GCC) as the co-expression measurement with R package rsgcc. For each lincRNA, protein coding genes with GCC >= 0.9 were taken as the "neighbors" with strong expression correlation. GO enrichment analysis for "neighbors" was implemented by R package GOstats [43]. Raw p-values were adjusted for multiple testing using BH procedure [44]. Significantly enriched terms (adjusted p-value <= 0.01) were assigned to the lincRNA.

To get a broad overview, fine-grained terms were further projected onto generic GO slim terms: Firstly, we downloaded GO slim file from http://www.geneontology.org/GO_slims/goslim_generic.obo, then mapped each GO term to its ancestor GO slim terms using map2slim in Perl package go-perl. After mapping lincRNA to GO slim terms, we further tested the enrichment of each lincRNA set for each GO slim term. For example, if we intended to see whether lincRNAs expressed in hES are biasedly enriched in GO slim term X, we classified all lincRNA in two ways: 1) expressed in hES or not expressed in hES, 2) enriched in term × or not enriched in term X. After Fisher's exact tests, GO slim terms with BY adjusted [45] p-value <= 0.05 and odds ratio > 1 were determined as significantly positively associated with hES lincRNA.

We also tried Pearson correlation coefficient instead of GCC, and changed the cutoff 0.9 to 0.85 and 0.95 for both measurements. Different parameters resulted in similar results, showing the robustness of our analysis pipeline.

### Transposable element (TE) content analysis

We downloaded annotations of repeats (hg19 rmsk table) from the UCSC Table Browser. Satellite, low complexity, and simple repeats were then excluded [28]. We compared genome coordinates of repeats with genome coordinates of lincRNA exons.

### Evolutionary dating of lincRNA

Firstly, we mapped human lincRNAs onto genomes of other species according to genome alignments by using UCSC liftOver with default parameters. If there were genome alignments between specified species and human, covering > 80% base pairs of human lincRNA, the aligned region within the specified specie was taken as the homolog of this human lincRNA. Using this criteria, we have identified 5,526 (~99%) homologs for our lincRNA catalog (5,576 lincRNA) among the 6 genomes (chimp: 5,383; gorilla: 5,258; orangutan: 5,308; rhesus: 5,064; marmoset: 4,481; mouse: 2,601). We further checked the alignment identity for these homologs in each species, and found that 95% of them with identity > 96% in chimp, > 95% in gorilla, > 92% in orangutan, > 87% in rhesus, > 82% in marmoset, > 50% in mouse.

Secondly, we dated lincRNAs according to appearance of homologs using parsimony rules. Briefly, if there are homologs in each of the species in the specified evolutionary clade for a lincRNA and no homologs in all outgroups, the lincRNA is dated to the period from the time divergent from its closest outgroup to the born time of the most recent common ancestor of that clade.

## List of abbreviations used

lincRNA: long intergenic non-coding RNA; lncRNA: long non-coding RNA; hES: human embryonic stem cell; FPKM: fragments per kilobase of transcript per million mapped reads; GO: gene ontology; TE: transposable element; GCC: Gini correlation coefficient.

## Competing interests

The authors declare that they have no competing interests.

## Authors' contributions

XT carried out the study, analyzed the data, prepared datasets for the database, and drafted the article. MH took on part of the data analysis. YD constructed the web portal and drafted the corresponding part of the article. ZHL collected the RNA-seq raw data from public databases. LCR provided embryonic stem cell expertise. GG supervised the whole project, including planning the project, analyzing the data, interpreting the results and revising the article.

## Supplementary Material

Additional file 1 **Screening lincRNAs from UCSC and RefSeq noncoding gene models**. We extracted RefSeq and UCSC nocoding genes from UCSC genome browser and screened them for lincRNA using filters similar to [18]. Except 5,456 lincRNAs already annotated by Ensembl, 115 lincRNAs have been kept. Click here for file

Additional file 2 Descriptions of RNA-Seq samples. Click here for file

Additional file 3 **Heat map representation of CpG islands, occupancy of Pol2, H3K4me3, H3K4me2, H3K27me3, H3K9me3 around promoter, H3K36me3 and H3K27me2 within gene body, and expression level of lincRNAs in hES cells**. The heat map is rank-ordered by FPKM of genes. The enrichment of Pol2, H3K4me3, H3K4me2, H3K27me3, H3K9me3, H3K36me3 and H3K27me2 was determined by ChIP-seq. All average binding is measured by −10*log_10 _(peak P-value) and is shown by color scale. The following color scales (white, no enrichment; blue, high enrichment) are used for Pol2, H3K4me3, H3K4me2, H3K27me3, H3K9me3, H3K36me3, H3K27me2, respectively. The density of CpG islands is displayed in color (blue, high density; white, absent). Occupancy of Pol2, H3K4me3, H3K4me2, H3K27me3, H3K9me3 are shown around gene TSS (upstream 5kb, downstream 5kb). Occupancy of H3K36me3 and H3K27me2 are shown within gene body for the major isoform in hES(the distances to TSS were normalized by major isoform transcript length for each gene). The right most column is the log_10_(FPKM+0.001) of genes. The red horizontal line separates genes which expressed in hES (FPKM>1) with those not. Click here for file

Additional file 4 **Novel lincRNA identification pipeline from RNA-seq**. We mapped reads onto hg19 using TopHat [39] and assembled transcript using Cufflinks [17]. We filtered assembled transcripts for lincRNA using filters similar to [18]. Suspicious transcripts with low expression level and few supporting reads for junctions were filtered out at last. Click here for file

## Declarations

Funding to pay the open access publication charges for this article was provided by State Key Laboratory of Protein and Plant Gene Research Open Funding.

This article has been published as part of *BMC Genomics *Volume 14 Supplement 5, 2013: Twelfth International Conference on Bioinformatics (InCoB2013): Computational biology. The full contents of the supplement are available online at http://www.biomedcentral.com/bmcgenomics/supplements/14/S5.
