# Supplementary figures and images for: Systematically profiling and annotating long intergenic non-coding RNAs in human embryonic stem cell
Source: BMC Genomics. 2013 Oct 16;14(Suppl 5):S3. doi: 10.1186/1471-2164-14-S5-S3 (PMC3852230; doi:10.1186/1471-2164-14-S5-S3)

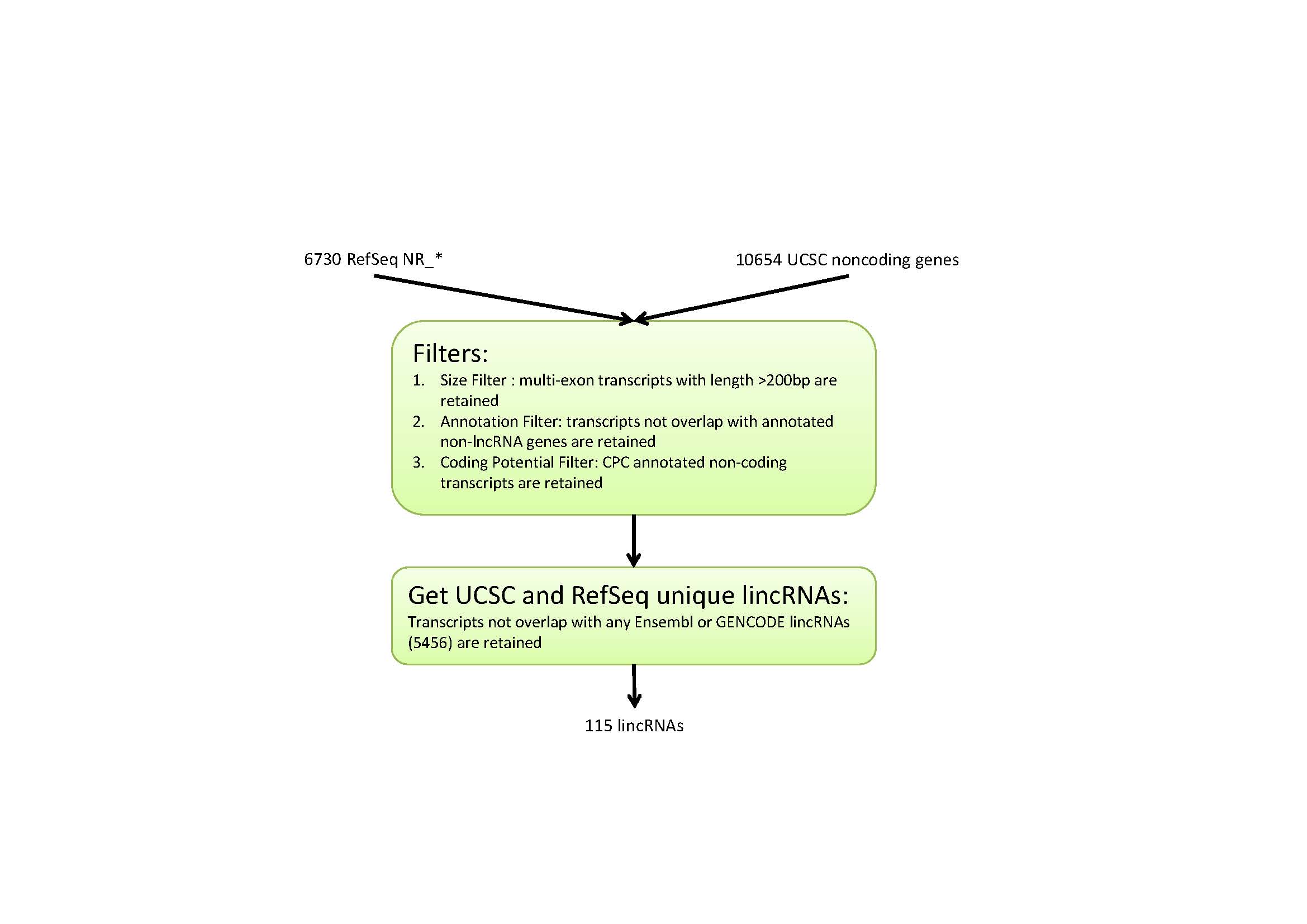

Supplement: Additional file 1 — Screening lincRNAs from UCSC and RefSeq noncoding gene models. We extracted RefSeq and UCSC nocoding genes from UCSC genome browser and screened them for lincRNA using filters similar to [18]. Except 5,456 lincRNAs already annotated by Ensembl, 115 lincRNAs have been kept. [file 1471-2164-14-S5-S3-S1.jpg]

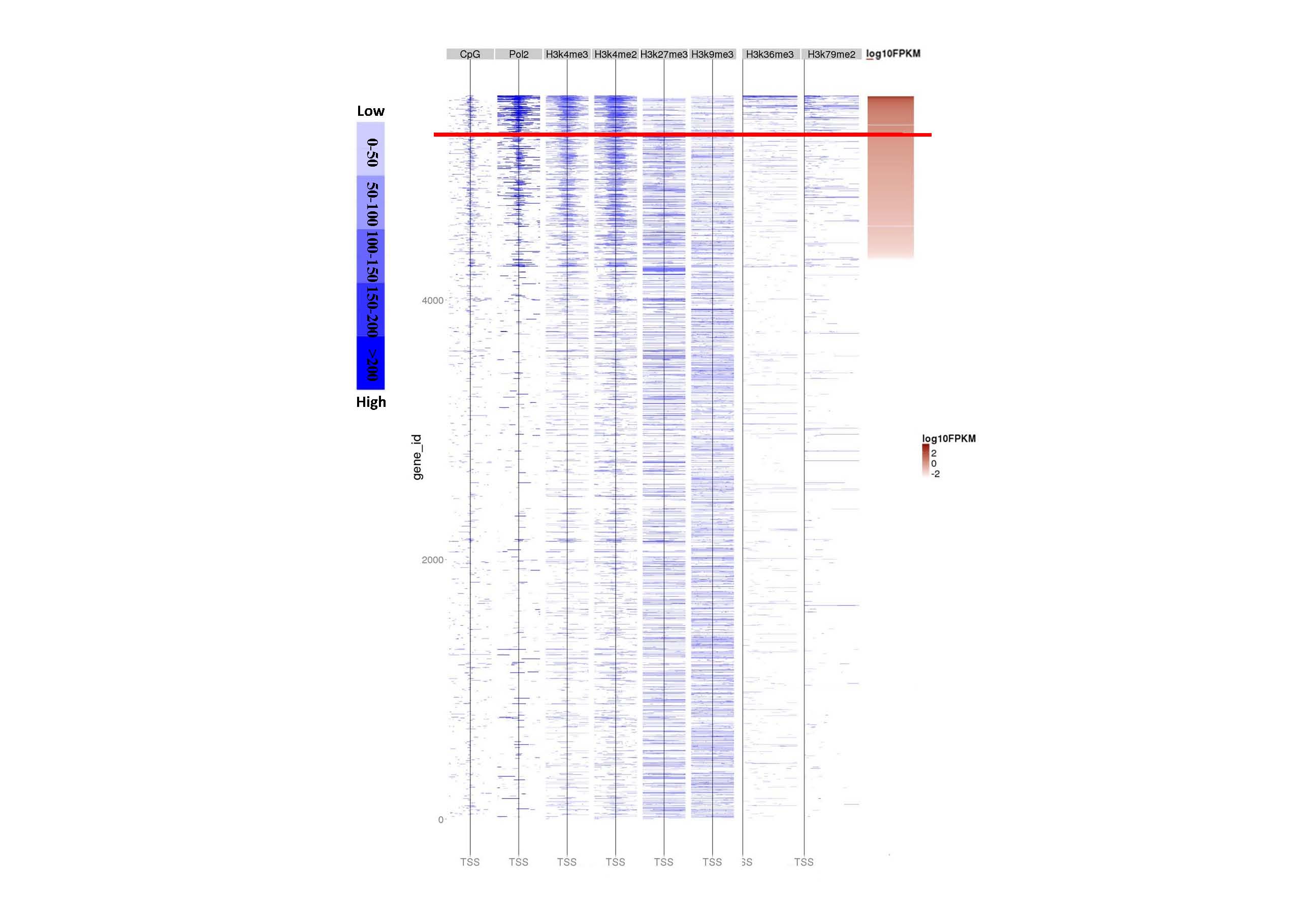

Supplement: Additional file 3 — Heat map representation of CpG islands, occupancy of Pol2, H3K4me3, H3K4me2, H3K27me3, H3K9me3 around promoter, H3K36me3 and H3K27me2 within gene body, and expression level of lincRNAs in hES cells. The heat map is rank-ordered by FPKM of genes. The enrichment of Pol2, H3K4me3, H3K4me2, H3K27me3, H3K9me3, H3K36me3 and H3K27me2 was determined by ChIP-seq. All average binding is measured by −10*log10 (peak P-value) and is shown by color scale. The following color scales (white, no enrichment; blue, high enrichment) are used for Pol2, H3K4me3, H3K4me2, H3K27me3, H3K9me3, H3K36me3, H3K27me2, respectively. The density of CpG islands is displayed in color (blue, high density; white, absent). Occupancy of Pol2, H3K4me3, H3K4me2, H3K27me3, H3K9me3 are shown around gene TSS (upstream 5kb, downstream 5kb). Occupancy of H3K36me3 and H3K27me2 are shown within gene body for the major isoform in hES(the distances to TSS were normalized by major isoform transcript length for each gene). The right most column is the log10(FPKM+0.001) of genes. The red horizontal line separates genes which expressed in hES (FPKM>1) with those not. [file 1471-2164-14-S5-S3-S3.jpg]

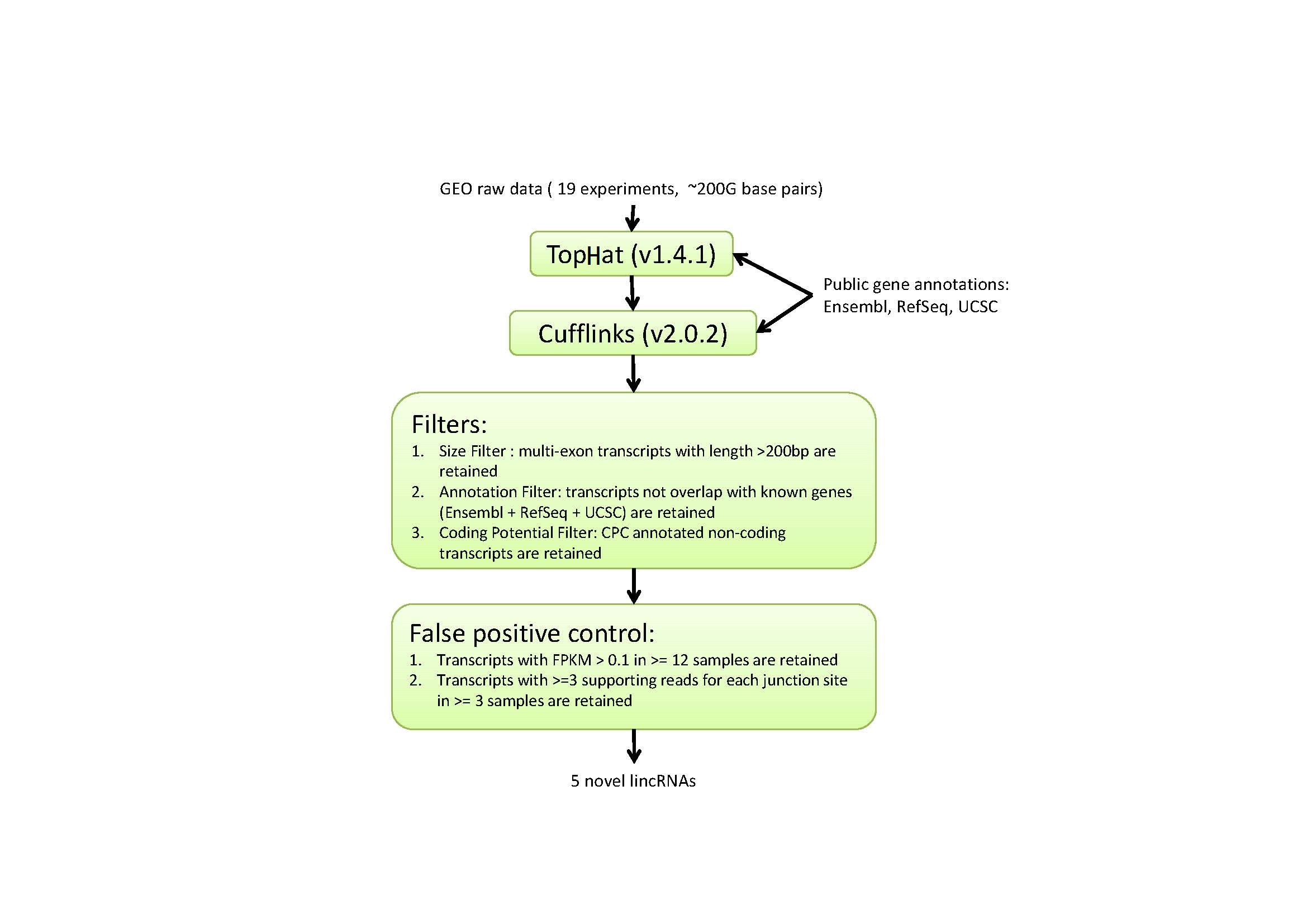

Supplement: Additional file 4 — Novel lincRNA identification pipeline from RNA-seq. We mapped reads onto hg19 using TopHat [39] and assembled transcript using Cufflinks [17]. We filtered assembled transcripts for lincRNA using filters similar to [18]. Suspicious transcripts with low expression level and few supporting reads for junctions were filtered out at last. [file 1471-2164-14-S5-S3-S4.jpg]
